# The circadian stimulus-oscillator model: Improvements to Kronauer’s model of the human circadian pacemaker

**DOI:** 10.3389/fnins.2022.965525

**Published:** 2022-09-27

**Authors:** Mark S. Rea, Rohan Nagare, Andrew Bierman, Mariana G. Figueiro

**Affiliations:** Light and Health Research Center, Department of Population Health Science and Policy, Icahn School of Medicine at Mount Sinai, New York, NY, United States

**Keywords:** circadian rhythms, circadian phase, modeling, van der Pol oscillator, light

## Abstract

Modeling how patterns of light and dark affect circadian phase is important clinically and organizationally (e.g., the military) because circadian disruption can compromise health and performance. Limit-cycle oscillator models in various forms have been used to characterize phase changes to a limited set of light interventions. We approached the analysis of the van der Pol oscillator-based model proposed by Kronauer and colleagues in 1999 and 2000 (Kronauer99) using a well-established framework from experimental psychology whereby the stimulus (S) acts on the organism (O) to produce a response (R). Within that framework, using four independent data sets utilizing calibrated personal light measurements, we conducted a serial analysis of the factors in the Kronauer99 model that could affect prediction accuracy characterized by changes in dim-light melatonin onset. Prediction uncertainty was slightly greater than 1 h for the new data sets using the original Kronauer99 model. The revised model described here reduced prediction uncertainty for these same data sets by roughly half.

## Introduction

The timing of our behavior and physiology is regulated by internal clock mechanisms. These various rhythmic behavioral (e.g., sleep) and physiological (e.g., cortisol) responses will cycle at approximately 24 h and are known as circadian (approximately daily) rhythms. These rhythms are, in part, regulated by different peripheral clocks in various organs or neural structures. These peripheral clocks each have a different intrinsic period and would be, therefore, asynchronous without a master clock that orchestrates them all so that our behavior and physiology work in concert. Indeed, the master clock located in the suprachiasmatic nuclei (SCN) sends neural signals to many of these peripheral clocks which in turn initiate their own neural or hormonal signals that are received by other organs or neural structures (Heyde and Oster, 2019).

**FIGURE 1:**
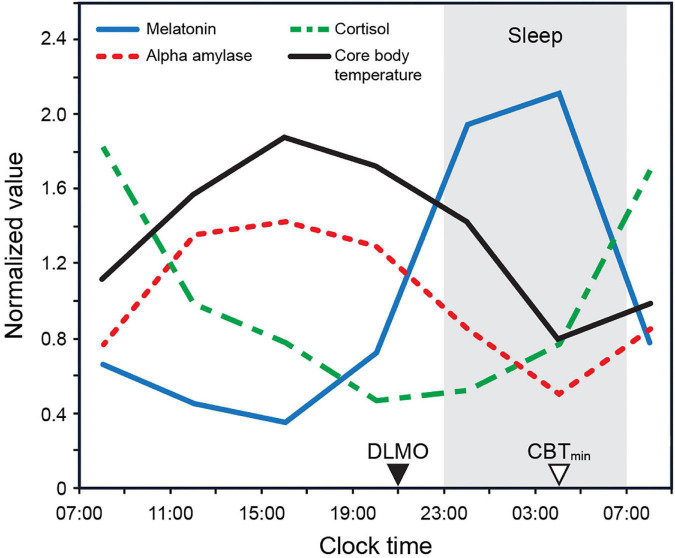
Biomarker 24 h profiles (normalized) for melatonin, cortisol, and alpha amylase as measured by Figueiro and Rea (2010) under constant dark conditions. The biomarker profile for core body temperature (CBT) adapted from Rüger et al. (2005). CBT_min_ = minimum core body temperature; DLMO = dim light melatonin onset.

For instance, the phase changes in the core body temperature (CBT) are likely modulated by a rhythmic input from the SCN acting upon the thermoregulatory centers within the hypothalamus, in turn modulating the set point and altering the thresholds for sweating and cutaneous vasodilatation (Krauchi, 2002). In humans, a circadian rhythm of heat loss from the distal limbs is evident, wherein the daily profiles of skin temperature and blood flow in these regions peak in the late evening before gradually declining to reach minima in the morning (Figure 1; Aschoff et al., 1972; Krauchi and Wirz-Justice, 1994). Similarly, the SCN closely regulates melatonin synthesis by the pineal gland (Arendt, 1995). Melatonin levels are high during the night and low during the day in both nocturnal and diurnal animals (see Figure 1). Melatonin down-regulation acts as an extension of the master clock, signaling circadian phase (Lewy, 1999b; Lewy et al., 1999).

This orchestration of peripheral clocks by the SCN enables us to perform complicated functions at the correct, coordinated times. Indeed, these clocks are necessary for survival. However, without some consistent, repetitive external timing stimulus, or *zeitgeber*, the SCN would “free run” and every person in society would perform their behavioral and physiological functions at different times. Prey and predators would be unpredictably available, as would reproductive partners. Indeed, without synchronized master clocks, it would be difficult for us to survive as a species. The primary *zeitgeber* for all terrestrial species is sunrise and sunset, that is, the natural 24-h light-dark cycle.

The intrinsic period of the SCN varies across individuals (Duffy et al., 2011). For humans spending most of their time outdoors, these various intrinsic periods will be naturally synchronized by the 24-h light-dark cycle entering the eyes. Indeed, a robust, 24-h light-dark cycle is the primary *zeitgeber* for human master clocks (Czeisler and Klerman, 1999). For humans who do *not* spend most of their time outdoors—that is, for nearly 90% of us (Klepeis et al., 2001)—light-dark exposure patterns can vary considerably, thus leading to disrupted behavior and physiology that can be asynchronous with other people in the same location. Modeling how light and dark affect circadian timing, particularly irregular or aperiodic patterns of light and dark, is important clinically and organizationally (e.g., the military) because “circadian disruption” can compromise health and performance.

To model changes in circadian phase, it is necessary to quantitatively address three domains. First, the light *stimulus* (S) for regulating the timing, or phase of the master clock must be defined. The master clock in humans, itself, does not receive direct light stimulation. The actual neural stimulus to the master clock must be processed by the retinae, converting photons into neural signals that are then conducted to the SCN via the retinohypothalamic tract (RHT). The retina is a complicated structure, combining photoreceptor responses into different neural pathways, one of which reaches the SCN. Thus, a model of retinal phototransduction will aid in a more accurate model of the stimulus (S) as it affects the master clock.

Second, it is important that the phase *response* (R) of the SCN to the light stimulus is measured. Since, however, it is not possible to record the human SCN responses *in situ*, we must rely on downstream measures of circadian phase, such as changes in dim light melatonin onset (Δ DLMO) or changes in minimum core body temperature (Δ CBT_min_) (Duffy et al., 1999; Benloucif et al., 2005). To have a more accurate estimate of the SCN phase response, other collateral inputs to these downstream measures must be considered. Ideally, these collateral inputs can be controlled or eliminated experimentally, but this is not always possible, particularly if light on the retina affects the phase response through a channel distinct from that which stimulates the master clock.

Between the stimulus and response is the *organism’s* (O) clock mechanism, which is the central focus of the present modeling exercise. Specifically, we have examined how different models of O affect the predicted relationship between S and R. Parsimony was one of our guiding principles in developing a model of O, eschewing “curve-fitting” parameters that might be used to produce incrementally better predictions. Another principle is convergence with known neurophysiology wherever possible, again eschewing “curve-fitting” parameters that cannot be linked to a known mechanism or neural structure. Naturally too, the uncertainties, both random and systematic, associated with measurements of both the S and the R in the various studies need to be considered when modeling the clock mechanism.

### Characterizing the stimulus

For an empirical assessment of predicted phase changes by the different models of the master clock, it is necessary to define the spectral and absolute sensitivities of the human retina to light as it signals photic information to the SCN. Surprisingly, however, little attention has been given to these critical stimulus aspects when evaluating the predictive capabilities of phase response models. A valuable characterization of the spectral and absolute sensitivities of the circadian phototransduction circuit in the human retina as it stimulates the SCN, both mathematically (Rea et al., 2021a) and neurophysiologically (Rea et al., 2021b), is provided by Rea et al.

**FIGURE 2:**
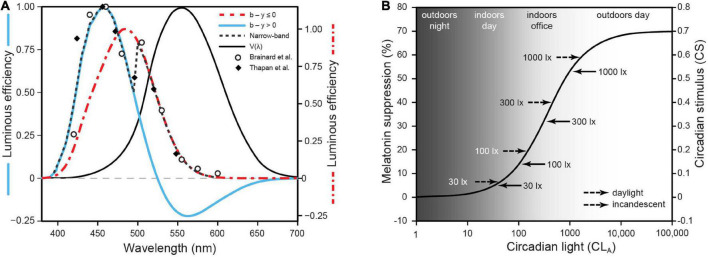
**(A)** The relative sensitivity of different narrowband light sources for suppressing nocturnal melatonin from Brainard et al. (2001) and Thapan et al. (2001). Also shown are the predictions from the two-state circadian phototransduction model (Eq. A1.1 from Supplementary Appendix 1), at 300 scotopic lux on the retina (Rea et al., 2021a,b). **(B)** The absolute sensitivity for the human circadian system as characterized by light-induced nocturnal melatonin suppression.

In terms of spectral sensitivity, it has been shown that the photopic luminous efficiency function [V(λ)] used to define “light” in commercial lighting is inappropriate for characterizing “light” for the circadian system (Tekieh et al., 2020). V(λ) represents the combined action spectrum of the middle-wavelength (M) cone sensitivity and the long-wavelength (L) cone sensitivity of the human macula, peaking at 555 nm. Brainard et al. (2001) and Thapan et al. (2001) independently showed that the peak spectral sensitivity of the circadian system, as measured by acute melatonin suppression is at or near 460 nm. There is no single photoreceptor that peaks at 460 nm as shown, with V(λ), in Figure 2A. Of particular note in this regard, the intrinsically photosensitive retinal ganglion cells (ipRGCs; Berson et al., 2002), the axons which form the RHT connecting the retina to the SCN, cannot account for the peak spectral sensitivity at 460 nm because the *in vivo* ipRGC photopigment, melanopsin (Provencio et al., 1998), exhibits an action spectrum peaking at or near 490 nm (after being filtered by the crystalline lens). Rea et al. (2021a,b) utilized *all* five retinal photoreceptors, together with a neural circuit consistent with orthodox retinal neurophysiology, to provide an more accurate, but non-linear characterization of the spectral sensitivity of the circadian system. Importantly, the non-linear aspects of the model requires different responses by the circadian phototransduction circuit in the retina for polychromatic sources than for narrowband light sources, like those employed by Brainard et al. (2001) and Thapan et al. (2001). Model predictions of spectral sensitivity at one scotopic (rod spectral sensitivity) light level for both narrowband and polychromatic sources are shown in Figure 2A.

Several other aspects of the modeled spectral sensitivity are worth noting. First, the blue versus yellow (b-y) spectral opponent mechanism, underlying one channel of human color vision, is also an important element of the modeled retinal circadian phototransduction circuit. This spectral opponent mechanism results in a two-state model, one state for “cool” sources where the spectral power distribution of the source results in a channel response of b > y, and the other for “warm” sources where the same channel response is b ≤ y. Thus, there are two spectral sensitivity functions for polychromatic sources shown in Figure 2A. Second, subadditivity is a characteristic of the circadian phototransduction circuit for “cool” sources as illustrated by the negative lobe in Figure 2A. For example, the response of the circuit to a narrowband light source of 460 nm will be *reduced* if a 530 nm narrowband light source is *added* to the photic stimulus. Subadditivity, which has been shown in several studies (Figueiro et al., 2004, 2005, 2008; Lee et al., 2017) is a direct consequence of the response by the b-y spectral opponent mechanism where adding a “yellow” light to a “blue” light can make the light visually appear less bright and achromatic (neither blue nor yellow). Equation A1.1 from Supplementary Appendix 1 mathematically describes circadian-effective light (CL_A_ 2.0) based upon the modeled spectral sensitivity of the human circadian phototransduction circuit, which includes the b-y spectral opponent mechanism shared with the visual system.

In terms of absolute sensitivity, humans have a higher threshold for photic stimulus activating the SCN than nocturnal mammals. To model this behavior, Rea et al. (2021a,b) incorporated a known retinal mechanism called shunting inhibition (Paulus and Rothwell, 2016). The high threshold results from rod activation of a specific type of amacrine cell. The modeled electrical shunt limits direct depolarization of the ipRGC in response to photon absorption and, thereby, from sending any neural signals to the SCN. In contrast, rods provide direct input to the ipRGC in nocturnal rodents, resulting in high sensitivity to light of the circadian phototransduction circuit (Bullough et al., 2005). Another feature of the model is the compression of neural response to light at high levels. Figure 2B shows the model predictions for different light levels, ranging from outdoors at night, to residential interiors, to commercial interiors, to outdoors during the day as a function of optical radiation spectrally weighted by CL_A_ 2.0. Equation A1.2 in Supplementary Appendix 1 mathematically describes the circadian stimulus (CS) to the human SCN.

Most pacemaker models use photopic illuminance (lux) as input (S). In experiments where only one light source is used, inaccurate characterization of spectral sensitivity is not important because the relative effectiveness of different light sources is irrelevant. Thus, only the amount of light needed to stimulate the pacemaker is relevant, irrespective of the units used to characterize the photic stimulus. Even for those experiments that used different “white” polychromatic lights generated by commercial light sources, the error in characterizing the stimulus introduced by V(λ) is small, particularly when the amount of light is quite large with respect to dim baseline conditions. In other words, where “white” lights are used differences in their *relative* spectral power distribution are less important than differences in their *absolute* spectral power distribution. Where narrowband light sources are used, however, erroneous characterization of the spectral sensitivity of the system can lead to larger errors. For example, the efficacy of a “blue” light for stimulating the SCN can be several orders of magnitude greater than that of a “red” light of the same photopic illuminance at the eye. For the present discussion of different pacemaker models, a direct comparison is made between spectrally weighting the photic stimuli in terms of CL_A_ 2.0, the spectral sensitivity of the circadian phototransduction mechanism in the eye, and in terms of V(λ), the combined spectral sensitivity of the M- and L-cones.

Nearly all pacemaker models have found it necessary to introduce a function that compresses the raw spectrally weighted irradiance, usually photopic illuminance, as the S input. As shown in Figure 2B, CS is a biophysically grounded compressive function of spectrally weighted irradiance, CL_A_ 2.0. Thus, a separate, arbitrary compressive function of spectrally weighted irradiance [e.g., V(λ) or CL_A_] is obviated. Because CL_A_ 2.0 and CS are grounded in the neurophysiology of the retina, these characterizations of the photic stimulus (S) to the pacemaker (O) are inherently better than a pacemaker model that utilizes an arbitrary compressive function for photopic illuminance or even for irradiance spectrally weighted by a different function such as CL_A_.

### Characterizing the response

The daily rhythm of melatonin concentration is currently the most acceptable marker of circadian phase and is widely used because it can be reliably assayed in blood, saliva, or urine (Refinetti, 2016). Dim light melatonin onset (DLMO) usually gets precedence over other circadian phase markers because it exhibits relatively greater robustness in the wake of various external influences (Lewy, 1999a). For instance, too much carbohydrate intake can significantly affect CBT and heart rate rhythms (Krauchi et al., 2002). Inherent changes to CBT are further essential to trigger master clock mediated immune response to any external threats that may compromise immune health (Coiffard et al., 2021). Cortisol and CBT parameters can also be masked by sleep, stress, and activity (Lewy et al., 1999). On the other hand, melatonin concentration and secretion remain relatively uninfluenced by these factors (Mirick and Davis, 2008). This also accords greater reliability for melatonin, over other circadian markers, to track circadian phase position.

### Characterizing the master clock

#### Preliminary comments

A major goal of any model is to predict data that are not part of the model development. However, the accuracy of model predictions is not isolated to the master clock (O) alone. Since all the pacemaker models in the literature have used photopic illuminance as the photic stimulus (S), there is likely some inherent error in the prediction accuracy. Similarly, there are several phase markers to characterize the response (R) of the master clock. Some are more fraught with sources of error (e.g., minimum core body temperature [CBT_min_]) than others (DLMO), but no single, downstream outcome measure is without uncertainty. Therefore, to compare model predictions, it is necessary to rely on accuracy metrics that reflect not just the error associated with the model (O), but with the stimulus (S) and the response (R) as well.

A variety of metrics can be used to assess the accuracy of S-O-R model predictions, but two of particular importance, and utilized here, when possible, are (1) mean absolute error (MAE), which characterizes the variance in the actual values with respect to the modeled values and (2) the amount of data within an accuracy criterion. Regarding the latter metric, an accuracy criterion of 1 h was chosen, reflecting the current state of model development; no model to date has been able to predict all data within an accuracy criterion of 30 min, and all models have shown to predict all data within an accuracy criterion of 3 h.

#### Mathematical models to predict circadian phase from personal light exposure

Limit-cycle oscillator models of O in various forms have been used to characterize phase changes to a limited set of light interventions. As detailed in Supplementary Appendix 2, the pioneering work from Harvard University toward the end of the last century examining the effects of amount, timing, and duration of light exposure on both phase and amplitude of the circadian clock laid the foundation for predicting circadian phase using a van der Pol oscillator with higher order non-linearities (Jewett et al., 1999; Kronauer et al., 1999, 2000). The initial *direct-drive* pacemaker models (Jewett and Kronauer, 1998), wherein the S exerts a direct influence on the state variables of the oscillator, could only accurately describe the response of the human circadian system to extended (4–8 h) bright (∼10,000 lux) light stimuli. Kronauer et al. (1999, 2000) revised their original van der Pol oscillator (Kronauer, 1990) with the introduction of a dynamic stimulus processor (Process L) that intervenes between the S and its effect on the self-sustaining limit-cycle oscillator (Process P), to allow predictions of R without limiting predictions to the lighting conditions from the original experiments by Jewett et al. (1994, 1997), Boivin et al. (1996), Klerman et al. (1996), and Jewett and Kronauer (1998). Their model, first published in 1999 and supplemented in 2000, will be subsequently referred to as Kronauer99.

A simpler cubic model with the van der Pol oscillator was alternatively proposed by Forger et al. (1999) (Forger99). The Forger99 framework included the Process L from Kronauer99, and also the sensitivity modulator within the pacemaker framework, to characterize how the current phase of the circadian oscillator could affect its sensitivity to the photic drive emanating from Process L. Photopic illuminance was used to characterize the photic stimulus (S). Experimental data from the human phase response curve (PRC) experiment by Khalsa et al. (1997) involving 21 healthy adult subjects were used to assess the prediction accuracy of the Forger99 model. Briefly, Khalsa et al. (1997) assessed change in phase, pre- and post-stimulus, of the plasma melatonin rhythm (onset, offset, and mid-point) in healthy entrained adults over the course of two 27- to 65-h constant-routine (CR) dim-light protocols. Pre-stimulus CR protocol was followed by exposure to the experimental photic stimulus (three ∼5,000–10,000 lx bright white light pulses delivered via fluorescent lamps) over the course of three successive days, centered (length ∼ 6 h) around the CBT_min_. While predicting the circadian phase changes for Khalsa et al. (1997) data, the original Kronauer99 model and the Forger99 model reported an MAE of 0.67 and 0.77 h, respectively.

Prediction accuracy for the Forger99 model and the Kronauer99 model has also been compared using independent data sets. Recently, for an independent data set involving 7 days of ambulatory light data collected in 27 shift workers with high levels of circadian disruption, Huang et al. (2021) compared and reported similar prediction accuracy (MAE ≈ 1 h) for both the Kronauer99 model (error < 1 h in 60% subjects) and the Forger99 model (error < 1 h in 50% subjects). It is, however, important to note that the light sensing device in Huang et al. (2021) study was first, located on the wrist, which has been shown to be a less accurate representation of the corneal stimuli (Figueiro et al., 2013), and second, the light sensing devices lacked unique calibration for each device, leading to poor inter-device consistency of measurement. Figueiro et al. (2013) reported as much as 20% measurement variability for six commercially available devices (Actiwatch Spectrum). Using three independent data sets utilizing calibrated circadian light sensing devices (see section “S and R” for details), we also compared the accuracy in predicting circadian phase for the two models (Supplementary Appendix 3). It was found that the average MAE of 1.07 h in predicting Δ DLMO using the original Kronauer99 model was quite comparable to the average MAE of 1.03 h for the Forger99 model. The modeling exercise further revealed that the percentage of subjects with error < 1 h was 53 and 54% for the Kronauer99 and Forger99 model, respectively. Thus, it can be argued that the Forger99 model did not yield substantial improvements in prediction accuracy over the Kronauer99 model.

In 2007, based upon circadian phase change data (Δ DLMO) from studies involving subjects investigated in environments free of time cues and with scheduled bedrest/activity patterns (enforced rest to activity ratio of 1:2), St Hilaire et al. (2007) proposed another version of the Kronauer99 model, having added an independent, non-photic (sleep/wake drive and associated behaviors) to the pacemaker. The experimental data sets used to develop and validate the St Hilaire model involved circadian phase predictions for a blind person (no visual perception but stable entrainment to scheduled 24-h sleep-wake cycle), CR protocols, forced desynchrony protocols (e.g., day length ∼ 20 h or 28 h), and exposures to bright light (5,000–10,000 lx) as well as dim light (1.5 lx). Like the photic drive from Process L, the non-photic sleep/wake state drive is modulated by a separate sensitivity modulator within Process P, which characterizes how the current phase of the circadian oscillator could affect its sensitivity to the sleep/wake state drive (Process N). Experimental data from the human phase response curve (PRC) experiment by Khalsa et al. (1997) (described above) were used to assess the prediction accuracy, wherein a decrease in the prediction accuracy was reported for the St Hilaire model (mean square error, MSE = 4.08; MAE not reported) as compared to the original Kronauer99 model (MSE = 3.82; MAE not reported).

Experimental circadian phase shifting data collected under dim-light protocol, as reported by Wright et al. (2001) was also used to assess the prediction accuracy of St Hilaire model. Briefly, Wright et al., conducted a study to determine whether a weak synchronizing stimulus (1.5 lx while awake, 0 lx while asleep) would entrain a subject experiencing an imposed daily cycle of 23.5, 24, or 24.6 h (total cycles ∼ 18–25). DLMO was tracked over the course of 40-h CR protocols, pre- and post-stimulus. A marginal increase in the prediction accuracy was reported for the St Hilaire model (mean square error, MSE = 0.91; MAE not reported) as compared to the original Kronauer99 model (MSE = 0.97; MAE not reported). More recently, for an independent data set involving up to 7 days of ambulatory measurements of light and activity recorded using wrist actigraphs, Stone et al. (2019a) reported a poorer accuracy for the original Kronauer99 model, as compared to the St Hilaire model, to predict circadian phase change (Δ DLMO) in day-shift working and night-shift working nurses. For the Kronauer99 model, Stone et al. (2019a) reported MAE of 0.65 ± 0.53 h (all ± represent standard deviation) on the diurnal schedule (error < 1 h in 64% subjects), and 1.19 ± 1.11 h on the night schedule (error < 1 h in 56% subjects). For the St Hilaire model comprising of both photic and non-photic (rest-activity pattern) inputs, Stone et al. (2019a) reported MAE of 0.69 ± 0.69 h on the day schedule (error < 1 h in 80% subjects), and 0.95 ± 0.77 h on the night schedule (error < 1 h in 68% subjects). However, an improved fit for the St Hilaire model was not evident for the Huang et al. (2021) data set mentioned above. In day-shift workers, Huang et al. (2021) reported MAE of 1.06 ± 0.95 h for the original Kronauer99 model (60% within ± 1 h), and 1.04 ± 0.78 h for the St Hilaire model (60% within ± 1 h). In night-shift workers, Huang et al. (2021) reported MAE of 3.72 ± 2.44 h for the original Kronauer99 model (15% within ± 1 h), and 3.70 ± 2.11 h for the St Hilaire model (7% within ± 1 h). Taken together, it can be concluded that the prediction accuracy is quite similar for the original Kronauer99 model and for the St Hilaire model across the two data sets. This suggests that non-photic input to O has little, if any, effect on accuracy of phase change predictions for those individuals with intact and functioning retinae.

The initial mathematical models developed and tested by independent research groups to predict circadian phase in humans have largely involved light-dark patterns as the primary *zeitgeber* input (Forger et al., 1999; Jewett et al., 1999). As discussed above, the comparisons pertaining to the St Hilaire et al. (2007) limit-cycle oscillator model suggests that non-photic components by themselves provide a much weaker entraining stimulus as compared to the light-dark patterns. However, some statistical models involved non-photic predictors such as sleep timing (Burgess et al., 2003), skin temperature (Kolodyazhniy et al., 2011), heart rate variability (Gil et al., 2013), and heart rate interbeat intervals (Gil et al., 2014). These additional predictors, however, are downstream responses (R) associated with circadian phase and not entraining stimuli (S) to the oscillator. In other words, these statistical models are essentially curve fits that do not adhere to the S-O-R paradigm proposed here. More importantly, they are unable to predict new data. Therefore, *post hoc* statistical models are not discussed further.

Worth mentioning, however, are the statistical modeling approaches involving supervised machine learning practices using a “black box” of artificial neural networks (ANNs) (Kolodyazhniy et al., 2012; Stone et al., 2019b). ANN models are inherently designed to learn complex, non-linear relationships between input (S) and output (R) data through curve fitting exercises utilizing a sigmoid function to characterize biophysical threshold and saturation values (DeLean et al., 1978). Studies by Kolodyazhniy et al. (2012) and Stone et al. (2019b), employed similar experimental protocols for day-shift workers, wherein 7 days of continuous ambulatory data were recorded with salivary DLMO tracked before and after the ambulatory wrist measurements in healthy adults. Using ANN models with the ambient blue light irradiance and skin temperature as the predictive inputs, the average MAE reported across the different diurnal data sets examined by Kolodyazhniy et al. (2012) and Stone et al. (2019b), was 1.11 ± 1.15 h, with the reported error < 1 h for about 68% of the population, which is comparable to the performance of the Kronauer99 model as reported in other data sets with similar demographics (Phillips et al., 2017; Woelders et al., 2017; Stone et al., 2019a).

A direct comparison of the circadian phase prediction accuracy of the original Kronauer99 model and a prominent ANN model (Hannay et al., 2019), which has evolved from the Forger99 model, was performed by Huang et al. (2021) using independent data collected in an ambulatory setting (7 days of light exposure and activity measurements in shift workers using wrist-worn devices). In day-shift workers, Huang et al. (2021) reported MAE of 1.06 ± 0.95 h for the original Kronauer99 model (60% within ± 1 h), and 1.49 ± 0.85 h for the ANN model (30% within ± 1 h). In night-shift workers, Huang et al. (2021) reported MAE of 3.72 ± 2.44 h for the original Kronauer99 model (15% within ± 1 h), and 3.81 ± 2.44 h for the ANN model (22% within ± 1 h). Overall, there is not enough evidence to date that the ANN modeling approach improves prediction accuracy over the original Kronauer99 model. It is also important to note that even though ANN models can be used to predict new data, they are not inherently grounded in circadian neurophysiology.

In summary, given the range of conditions under which pacemaker models have been tested, and the consistency in its predictive ability across populations either stably entrained or with circadian misalignment, the oldest, Kronauer99 oscillator model remains as good as any. For these reasons, the prediction accuracy of the Kronauer99 model was used as the benchmark for subsequent modeling exercises, as has been done by most studies before. A major advantage of the modeling exercise performed and reported below is the stimulus (S) and the response (R) used. The variable CL_A_/CS, grounded in retinal neurophysiology and neuroanatomy, is used to provide input to the SCN and Δ DLMO is used as a marker of circadian phase.

## Revised predictions of circadian phase

### Evaluating pacemaker model predictions using four independent data sets

The data sets listed in Table 1 have some advantages for evaluating pacemaker (O) model predictions of circadian phase change (R) due to light stimulation (S). First, and foremost, a useful pacemaker model should be able to predict data that were not used in model development. All four studies introduced here measured phase changes resulting from photic stimulation and have not been used in any pacemaker model development. Second, there are four independent data sets, not just one. So, pacemaker model predictions can be independently tested and compared, thus avoiding the possibility of “getting lucky” in predicting just one data set. Third, all four data sets were obtained under field conditions, not under controlled experiments. As such, these data should be inherently more variable than laboratory data because light exposures, as well as other potential influences on circadian phase, were not controlled. If pacemaker models can predict phase changes for ambulatory data, they should also be able to predict phase changes in controlled laboratory experiments. Therefore, these four data sets represent a “worst-case” test of pacemaker models. Fourth, the proper specification of S and R is critical in testing pacemaker models. Without confidence in S and R, model prediction will be compromised. These four data sets are unique in this regard because the personal light sensors have been calibrated in terms of CL_A_, not photopic illuminance, and because assessments of the phase maker, DLMO, were obtained by a known, carefully supervised bioassay laboratory. Finally, we are intimately familiar with the design and execution of these experiments so are confident that we have good measures of the S and R data.

**TABLE 1:** An overview of the studies that provided the data sets for modeling.

Data set	Sample characteristics /Position of light sensor	Protocol
Appleman et al., 2013	*n* = 21 adults age = 22.5 ± 3.9 years 11 females Device: Daysimeter-D Position of Daysimeter device: Wrist	DLMO assessed on the last night of the 5-day baseline period and on the last night of the intervention week. For the intervention, subjects were either assigned to an advance group receiving 2 h of LED blue light (λ_max_ ≈ 476 nm) exposure in the morning and 3 h of orange-filtered light (λ < 525 nm = 0) in the evening, or a delay group receiving the blue light for 3 h in the evening and 2 h of orange-filtered light in the morning. Subjects were required to follow a 90-min advanced sleep schedule while wearing a calibrated wrist-worn Daysimeter.
Sharkey et al., 2011	*n* = 25 adults with delayed sleep age = 21.8 ± 3.0 years 13 females Device: Daysimeter-S Position of Daysimeter device: Headset (at eye level)	Examined the effects of an advanced sleep/wake schedule and morning blue light on circadian phase in adults with late sleep schedules and subclinical features of delayed sleep phase syndrome (DSPD). Subjects were required to follow a fixed, individualized, advanced (1–2.5 h) sleep/wake schedule that included 7.5 h of time in bed per night. DLMO assessed on the last night of the baseline week and on the last night of the intervention week. Following baseline, subjects were assigned to either receive LED blue light (λmax ≈ 470, ∼225 lux, *n* = 12) or “dim” (<1 lux, *n* = 13) light for 1 h after waking each day. Light exposures were tracked from wake to sleep using head-worn Daysimeters.
Rea et al., 2016	*n* = 11 adults Age = 25.4 ± 6.9 years 7 females Device: Daysimeter-S Position of Daysimeter device: Attached to collar	DLMO was assessed on the last night of the 2-week baseline collection period and again on the last night of the 2-week intervention collection period. Active light intervention comprised of LED blue light goggles (CS = 0.5) worn every morning for a minimum of 2 h or a maximum of 4 h depending upon previous light exposure. Orange-filtered glasses (λ < 525 nm = 0) were worn during the evening hours from 5:00 pm to bedtime. Daysimeters as well as wrist-worn actigraphs were continuously worn all 4 weeks.
Figueiro et al., 2014	*n* = 23 regular adults Age = 31.1 ± 11.1 years 17 females Device: Daysimeter-D Position of Daysimeter device: Wrist	All the subjects experienced an advance protocol (receiving 2 h of blue light exposure in the morning and 3 h of orange-filtered light in the evening), as well as a delay protocol (blue light for 3 h in the evening and 2 h of orange-filtered light in the morning), in a counter-balanced order. Subjects were required to follow a 90-min advanced sleep schedule (except for the baseline period) while wearing a calibrated wrist-worn Daysimeter. For both sessions, DLMO assessed on the last night of the 5-day baseline period and on the last night of the intervention week.

### S and R

All four ambulatory experiments utilized in-house, calibrated personal light sensors – Daysimeters, to quantify S (Rea et al., 2008, 2011; Figueiro and Rea, 2010; Miller et al., 2010; Sharkey et al., 2011) and in-house, well-controlled bioassay assessments of light-induced DLMO changes to quantify R. There are different versions of the light sensor (Daysimeter) as described in detail in Figueiro et al. (2013). These Daysimeters have been used to study circadian phase disruption in several sample populations: (1) nurses (Rea et al., 2008; Miller et al., 2010), (2) school children (Figueiro and Rea, 2010), (3) school teachers (Rea et al., 2011), (4) young adults (Sharkey et al., 2011), and (5) older adults Figueiro et al. (2013). Broadly, a Daysimeter contains a red-green-blue (RGB), solid-state photosensor package, with an infrared (IR) filter. The R, G, and B reading from a calibrated light source enable software to compute a variety of spectrally weighted irradiance values such as photopic illuminance (lux), CL_*A.*_ and CS. These sensors also contain a three-axis, solid-state accelerometer package to measure a behavioral response (R) influenced by the circadian system, called Activity Index (AI), simultaneous with the light stimulus (S) (Bierman et al., 2005). Sampling rates were once every second, while storage rates (average values) varied between once every 30 s to once every 180 s (depending upon the study). The lab maintains a calibration file for each of the units developed. Importantly, not all photosensors used in other experiments are known to have been calibrated, and those that have been calibrated do not necessarily provide circadian-relevant values of light, like CL_A_ or CS. Again, this failure to calibrate light sensors for the purposes of assessing pacemaker models will reduce accuracy of estimates.

In terms of R, Δ DLMO is a considered to be the best measure of circadian phase change (Lewy, 1999a). A trained nurse collected and stored all samples for biomarker assessments. Table 1 summarizes the four data sets used in our assessments of model prediction accuracy.

Two points should be noted regarding the light measurements in these four studies. First, the Daysimeter devices used to record circadian light exposures (CL_A_/CS) across the four datasets from Table 1 were calibrated based upon the original Rea et al. model of human circadian phototransduction (Rea et al., 2005, 2012), and not based upon the most recent revision to the model characterizing circadian light exposure in units of CL_A_ 2.0/CS (Rea et al., 2021a,b). Second, for the Appleman et al. (2013) dataset, only circadian light exposures (CL_A_) were recorded, and hence, any prediction accuracies using photopic light exposures as light stimuli could not be reported.

### Assessing prediction accuracy

We used a six-step, serial approach, outlined below, to evaluate model prediction accuracy.

Step 1: Determine whether predictions are better with or without Process L for the original limit-cycle oscillator model.

Step 2: Determine whether CL_A_ is better than photopic illuminance for prediction accuracy.

Step 3: Determine whether CS as photic input obviates Process L.

Step 4: Determine whether considering only the morning light exposure period can improve prediction accuracy.

Step 5: Determine the importance of the sensitivity modulator and the initial estimate of the clock time for the CBT_min_.

Step 6: Determine if the original Kronauer99 model parameters still hold given the change in the characterization of the light stimulus input.

It should be noted that Δ DLMO was used in all four studies, so it was not possible to assess how other downstream outcome measures, R, might contribute to prediction accuracy.

#### Model evaluation

The Kronauer99 model was numerically solved and propagated in time using the parameter values published in Kronauer et al. (2000) and as reported below in Table 2 (column 2). Since the outcome measure for the Kronauer99 model consists of CBT_min_ and not DLMO, prior biomarker data from our lab (Figure 1) were used to establish the relationship between CBT_min_ and DLMO (DLMO = CBT_min_ – 7 h). Constant routine (CR) estimates of initial circadian phase were not available for any of the four field studies. Even though the model at baseline was initialized assuming a CBT_min_ of 0400 across the groups, a systematic analysis was later performed by varying and optimizing the initial CBT_min_ at baseline from 0300 to 0900 in increments of 1 h. Predicted CBT_min_ at the end of the baseline period was used as the initial CBT_min_ for analyzing phase changes following the intervention period for each participant. For the analyses reported below, calibrated and personalized light-exposure data recorded for each individual participant across four field studies (Table 1) were used as the photic input to predict changes in circadian phase and validated against the proxy for Δ CBT_min_ (Δ DLMO). Error in prediction accuracy, calculated at an individual level, was subsequently averaged across individuals within the group and these values are reported in Tables 3–7, for each of the four datasets.

**TABLE 2:** Published parameter values from Kronauer et al. (2000) and the range of parameter values over which prediction accuracy of the modified model with CL_A_ and CS as inputs was re-evaluated.

Parameters	Published values	Range
α_0_ (Process L)	0.05	0.01–0.19
β (Process L)	0.0075	0.0025–0.0200
G (Process L)	33.75	NA
p (Process L)	0.6*	0.1–1.0
I_0_ (Process L)	9500	NA
μ(Process P)	0.13	0.01–0.30
q(Process P)	0.33	0.15–1.00
k(Process P)	0.55	0.15–0.95

*It should be noted that we have used the Kronauer et al. (1999) value of *p* = 0.6; *p* = 0.5 in Kronauer et al. (2000).

**TABLE 3:** Summary of model predictions with and without Process L for the Kronauer99 model.

Model	Data set	*R* ^2^	Mean absolute error (MAE) in h	Subjects with error < 1.0 h (%)
Kronauer99 without Process L	Rea et al., 2016 Figueiro et al., 2014 Sharkey et al., 2011 **Average**	0.07 0.11 0.11 **0.10**	1.48 1.16 1.58 **1.41**	45 60 32 **46**
Kronauer99 with Process L	Rea et al., 2016 Figueiro et al., 2014 Sharkey et al., 2011 **Average**	0.11 0.42 0.21 **0.25**	0.91 0.86 1.43 **1.07**	55 67 36 **53**

Note that the data from Appleman et al. (2013) could not be used for this analysis.

**TABLE 4:** Summary of model predictions with and without Process L for the Kronauer99 model using CL_A_ as the photic stimulus.

Model	Data set	*R* ^2^	Mean absolute error (MAE) in h	Subjects with error < 1 h (%)
CL_A_ without Process L I_0_ = 9500	Rea et al., 2016 Figueiro et al., 2014 Sharkey et al., 2011 Appleman et al., 2013 **Average**	0.04 0.31 0.00 0.35 **0.18**	1.15 1.04 1.57 1.2 **1.24**	55 62 27 48 **48**
CL_A_ with Process L I_0_ = 9500	Rea et al., 2016 Figueiro et al., 2014 Sharkey et al., 2011 Appleman et al., 2013 **Average**	0.18 0.69 0.01 0.77 **0.41**	0.57 0.76 1.54 0.82 **0.92**	91 71 27 67 **64**

**TABLE 5:** Summary of model predictions for the Kronauer99 model using CS as photic input (CS-oscillator model).

Model	Data set	*R* ^2^	Mean absolute error (MAE) in h	Subjects with error < 1 h (%)
CS without Process L I_0_ = 0.7	Rea et al., 2016 Figueiro et al., 2014 Sharkey et al., 2011 Appleman et al., 2013 **Average**	0.34 0.73 0.16 0.77 **0.50**	0.92 1.20 0.94 1.22 **1.07**	64 49 59 38 **52**
CS with Process L I_0_ = 0.7	Rea et al., 2016 Figueiro et al., 2014 Sharkey et al., 2011 Appleman et al., 2013 **Average**	0.49 0.72 0.17 0.80 **0.55**	0.66 0.66 1.21 0.63 **0.79**	91 80 41 86 **75**

**TABLE 6:** Summary of model predictions for the CS-oscillator model with light exposures from only 0600 to 1000 considered.

Model	Data set	*R* ^2^	Mean absolute error (MAE) in h	Subjects with error < 1 h (%)
Morning CS (0600 – 1000)	Rea et al., 2016 Figueiro et al., 2014 Sharkey et al., 2011 Appleman et al., 2013 **Average**	0.29 0.31 0.01 0.06 **0.17**	1.76 1.31 1.93 1.56 **1.64**	27 42 18 48 **34**
All CS (24 h)	Rea et al., 2016 Figueiro et al., 2014 Sharkey et al., 2011 Appleman et al., 2013 **Average**	0.49 0.72 0.17 0.80 **0.55**	0.66 0.66 1.21 0.63 **0.79**	91 80 41 86 **75**

**TABLE 7:** Summarizing the effect of changing CBT_min_ on prediction accuracy for the CS-oscillator model across the four data sets.

Data set	CBT_min_ (time)	*R* ^2^	Mean absolute error (MAE) in h	Subjects with error < 1 h (%)
** Prediction accuracy with base case CBT_min_**				
Rea et al., 2016	0400	0.49	0.66	91
Figueiro et al., 2014	0400	0.72	0.66	80
Sharkey et al., 2011	0400	0.17	1.21	41
Appleman et al., 2013	0400	0.80	0.63	86
**Average**	—	**0.55**	**0.79**	**75**
Prediction accuracy with optimum CBT_min_*				
Rea et al., 2016	0300	0.52	0.60	91
Figueiro et al., 2014	0500	0.73	0.60	82
Sharkey et al., 2011	0900	0.27	0.59	91
Appleman et al., 2013	0400	0.80	0.63	86
**Average**	—	**0.58**	**0.61**	**88**

*Primary consideration while determining the optimum CBT_min_ was % subjects with Error < 1 h.

All analysis was undertaken using MATLAB software (MathWorks, Natick, MA, United States), wherein the MATLAB^®^ numerical solver, *ode45*, was primarily used to solve the mathematical differential equations. (Reasonable requests to the corresponding author for additional information about the computations or individual data will be fulfilled.)

##### Step 1: Determine whether predictions are better with or without Process L for the original limit-cycle oscillator model

We investigated the impact of prior light exposures by including and excluding Process L in the working model framework for each of the data sets. For this analysis we simply bypassed Process L, inputting the light data into Process P directly. The light data from the Daysimeter has a relatively high temporal bandwidth being sampled at 3-min intervals or less. The importance of the high frequency content for accurate predictions helped us determine the importance of the temporal dynamics of Process L and values of the involved time constants in a subsequent analysis. For example, Process L does not treat each light stimulus to the pacemaker independently. Rather, the magnitude of the effect from a given light stimulus, depends upon the magnitude of the effect caused by previous light stimuli.

As is evident from Table 3, the exclusion of Process L from the original Kronauer99 model substantially increases the MAE from 1.07 to 1.41 h and the percentage of subjects with error < 1 h drops from 53 to 46%. Thus, Process L improves the circadian phase prediction accuracy. This means, in effect, that light exposures are not independent when driving the SCN and sampling intervals should be short (<180 s). Rather, one must know with relatively high precision the previous light exposure before the impact of the next light exposure can be predicted.

##### Step 2: Determine whether CL_A_ is better than photopic illuminance for prediction accuracy

For this step, CL_A_ replaced photopic illuminance as the input light parameter “*I*” as specified in the Kronauer99 framework (Eq. 6 in Supplementary Appendix 1). The rest of the model parameters were maintained. Comparing the results from Tables 3, 4, using CL_A_ as input to Process L improves the MAE from 1.07 h (original Kronauer99 model) to 0.92 h and the percentage of subjects with error < 1 h increases from 53 to 64%. Thus, prediction accuracy improves by adjusting the spectral sensitivity of the retinal mechanisms providing input to the Kronauer99 model (i.e., substituting CL_A_ for photopic illuminance).

##### Step 3: Determine whether circadian stimulus as photic input obviates Process L

For this step, CS replaced photopic illuminance as the input light parameter *“I”* as specified in the Kronauer99 framework (Eq. 6 in Supplementary Appendix 1). The value of *I*_0_ (Eq. 6 in Supplementary Appendix 1) was set to 0.7, which is the mathematical asymptote for CS as defined by Rea and colleagues (Rea et al., 2005, 2012, 2021a,2021b). CS is a sigmoid function, inherently rendering every weak light stimulus equal (below threshold) and every strong light stimulus equal (above saturation). If, for example, the light stimuli were always either above saturation or below threshold, characterizing light exposures in units of CS would reduce the significance of Process L for model predictions. Indeed, there is no difference between the original Kronauer99 model with photopic illuminance as the photic input to Process L (Table 3, MAE = 1.07 and a 1-h accuracy criterion = 53%) and using CS without Process L (Table 5, MAE = 1.07 and a 1-h accuracy criterion = 52%).

However, there is significant improvement when CS is used as photic input to Process L (Table 5), MAE = 0.79 and a 1-h accuracy criterion = 75%). Thus, correctly characterizing the photic input, in terms of both spectral (CL_A_) and absolute (CS) sensitivity over the full operating range of the retinal phototransduction mechanisms, to Process L improves model prediction accuracy.

##### Step 4: Determine whether considering only the morning light exposure period can improve prediction accuracy

Diurnal species, including humans, typically exhibit intrinsic periods slightly longer than 24 h. To entrain to local time, morning light exposure is particularly important because it will advance the clock phase and the majority of humans free run with a period slightly longer than 24 h. We examined whether measuring morning light exposure alone would accurately predict phase changes. Several permutations of using only the selective portions of the daily light-dark patterns to the predictive models were performed. As compared to continuous measurements of light exposure throughout the wake period as the predictive input, selective light pulse input models fared poorly. MAE and percent subjects with error < 1 h values for one such scenario wherein only the light-dark patterns from 0600 to 1000 were considered as the predictive inputs, are shown in Table 6. Adjusting the start (0600) and end times (1000), or splitting the exposure window (for e.g., 0600–0800 and 1800–2000), did not improve the prediction accuracy (data not reported).

##### Step 5: Determine the importance of the sensitivity modulator and the initial estimate of the CBT_min_

The Kronauer99 model included a sensitivity modulator between Process L and Process P which controls the relative effectiveness of photic exposures depending upon the circadian phase of the pacemaker at the time of exposure (Jewett et al., 1999). For example, a light exposure in the early morning should be more effective for inducing a phase change than that very same light exposure mid-day. Values generated by the sensitivity modulator depend upon the value of the circadian phase marker, CBT_min_. The circadian phase marker was initially estimated to occur at 0400 to depict a “typical” CBT_min_ for people entrained to the solar day; that is, 4 h after midnight. Importantly, this assumed value of 0400 also sets the values for the light drive terms, *xandx*_*c*_, quite apart from the sensitivity modulator (Supplementary Appendix 2).

**FIGURE 3:**
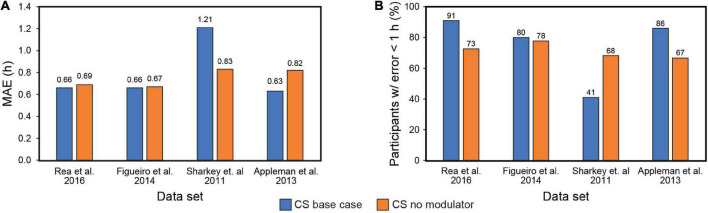
Mean absolute error (MAE) across the four data sets with (blue bars) and without (orange bars) the modulator **(A)**; Percent subjects with error < 1 h across the four data sets with (blue bars) and without (orange bars) the modulator **(B)**. MAE = mean absolute error. CS = circadian stimulus.

Our systematic review of Kronauer99 model components also included an examination of the role of the initial estimate of the CBT_min_ in prediction accuracy. Theoretically, a large enough baseline data, consisting of light exposure history and biomarker assessment, should obviate the initial CBT_min_ estimate, as subsequent predictions of post-intervention phase will utilize the CBT_min_ at the end of the baseline period as the initial phase. Figure 3 shows the results of including or excluding the sensitivity modulator with CBT_min_ = 0400 when using CS as the photic input (S) to Process L for the four data sets (see Table 5). Of note, it will be recalled that the four data sets employed DLMO as the phase marker, but the analysis is based upon light-induced changes in DLMO (i.e., ΔDLMO). Thus, the absolute values of DLMO are unimportant, and all phase changes are evaluated relative to the phase determined at the end of the baseline period. On average, MAE across the four data sets *dropped* from 0.79 to 0.75 h with exclusion of the sensitivity modulator. However, the percentage of subjects with error < 1 h *decreased* from 75 to 71%. A closer look at these two metrics for the individual data sets shows that including the sensitivity modulator *improved* both MAE and the percentage of subjects with error < 1 h for three of the four data sets. Indeed, the Sharkey data were significantly worse when the sensitivity modulator was included. These results suggested that the sensitivity modulator could not be examined alone, but rather that there was an interaction between the assumed CBT_min_ value and the sensitivity modulator in determining predictive accuracy of the model.

**FIGURE 4:**
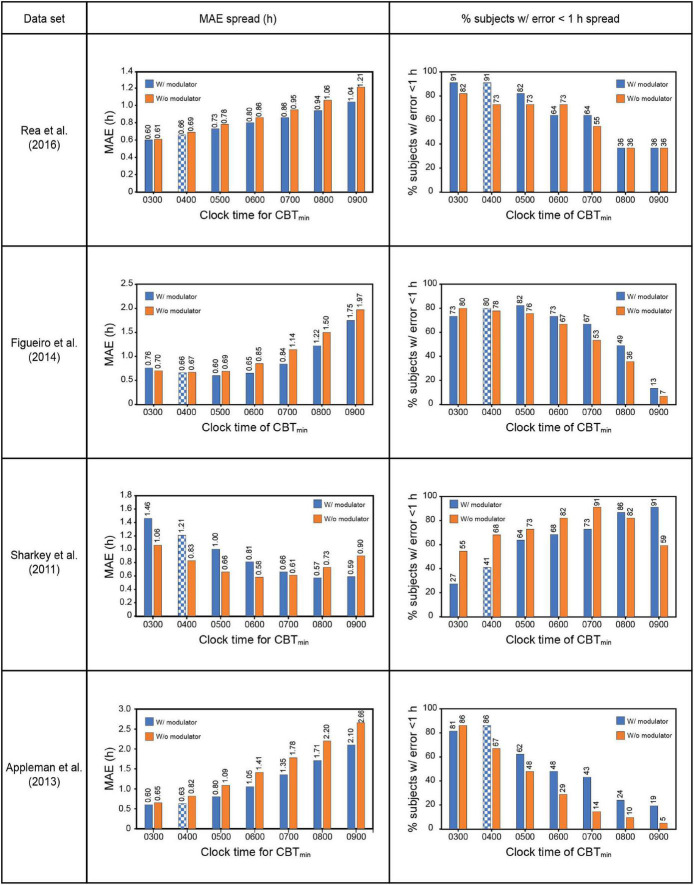
Effect of changing CBT_min_ on prediction accuracy across the four datasets. The checkered bars depict the assumptions in the original Kronauer model with the sensitivity modulator and an initial typical CBT_min_ = 0400. CBT_min_ = minimum core body temperature; MAE = mean absolute error.

The Sharkey data set involved young adult subjects with delayed sleep. This would suggest *a priori* that the CBT_min_ would be much later than the 0400 value estimated originally by Kronauer99. Whereas no other set of subjects would have been as phase delayed, it was deemed necessary to determine how the assumed value of CBT_min_ affected prediction accuracy. Given the absence of CR protocol estimated initial circadian phase, Figure 4 shows the prediction accuracy values where CBT_min_ was systematically varied between 0300 and 0900 for the four data sets. The checkered bars in Figure 4 depict the assumptions in the original Kronauer99 model with the sensitivity modulator and an initial CBT_min_ = 0400.

The prediction accuracy values in the upper half of Table 7 are the same as those in the lower half of Table 6 where the CBT_min_ = 0400 and the sensitivity modulator is included in the model. The lower half of Table 7 summarizes the findings from interaction plots in Figure 4. Specifically, inclusion of the sensitivity modulator *with* the optimum time of the initial circadian phase marker (i.e., the value of CBT_min_) *always* led to the greatest prediction accuracy. For each of the four data sets, the prediction accuracy values, MAE and percent subjects with error < 1 h, are shown with the optimum CBT_min_ *with* the sensitivity modulator. Assuming baseline CBT_min_ = 0400 was only accurate for one of the four data sets (Appleman et al., 2013) and as expected, the discrepancy between optimum CBT_min_ value and CBT_min_ = 0400 was greatest for the Sharkey data. Table 7 shows the importance of setting an optimum value for CBT_min_; MAE decreased from 0.79 to 0.61 and the percent of subjects with error < 1 h increased from 75 to 88%.

These results demonstrate that chronotype is important for accurately predicting light-induced phase changes, particularly for more extreme chronotypes like those in the Sharkey study. It will be recalled that Figueiro et al. (2014) employed both phase-advanced and phase-delayed chronotypes in their study. As might be expected, the optimum CBT_min_ for the delayed group (owls) was later than that for the advanced group (larks). Consistent with an initial estimate, CBT_min_ = 0400 was optimum for the larks, with a MAE of 0.72 h and 83% subjects with error < 1 h. The optimum CBT_min_ for the owls was 0500, with a MAE of 0.75 h and 86% subjects with error < 1 h. Despite their designated chronotype, the difference in CBT_min_ for the two groups was quite small, likely because all the people in this study worked during the day.

Broadly speaking, this serial analysis demonstrates that prediction accuracy can vary quite considerably depending upon the explicit or implicit assumptions about the light stimulus (S) and the study cohort (O). Therefore, the CS-oscillator model developed here and derived from the original framework of the Kronauer99 model is an improvement in prediction accuracy.

##### Step 6: Validate the original Kronauer99 model parameters given the change in characterization of the light stimulus input

Since the light stimulus (S) to the model developed here is different than that used by Kronauer99, it was necessary to examine how well their original model parameters (equations B2.1–B2.8, Supplementary Appendix 2) affected prediction accuracy. Their published values of their model parameters are shown in Table 2.

All model parameter values were systematically varied over a range as shown in Table 2 to determine how they might affect prediction accuracy from the model developed here for the four data sets. G is inherently derived from α_0_ and β, and hence was excluded from this parameter-validation analysis. The originally published I_0_ value of 9500 was preserved when the light stimulus was changed from photopic illuminance to CL_A_. However, when the light stimulus input was changed from CL_A_ to CS, the default value of I_0_ had to be changed to 0.7, which is the mathematical asymptote for CS as defined by Rea et al., 2005, 2012, 2021a,2021b. Being an asymptote, I_0_ optimization was also excluded from the parameter-validation analysis.

Following Kronauer99, the association between the light exposures recorded and the modeled time constants – α_0_ and β, from Process L, is governed by the parameter “*p*,” or the light exponent. Contour plots, as reported in Supplementary Appendices 4, 5, demonstrate how the changes in values of α_0_ and β affect the MAE and percent subjects with error < 1 h, respectively. A change in prediction accuracy as a function of changing *p*, the light exponent, is also reported. This analysis revealed that, with a few exceptions in the Sharkey et al. (2011) data set, the prediction accuracy for base case scenario (α_0_ = 0.05; β = 0.0075; *p* = 0.06) was always within ± 10% of the best achievable accuracy over the entire range of the parameters deployed across the data sets. In other words, there was no evidence that prediction accuracy could be substantially improved by changing the parameter values published in Kronauer99 and used in our analyses. Therefore, the model developed here retained those originally published for Process L.

Similarly, Supplementary Appendices 6, 7 examined the prediction accuracy for changes to the published Process P parameter values (μ = 0.13; *k* = 0.55; *q* = 0.33). Again, with a few exceptions in the Sharkey et al. (2011) data set, the original values were always within ± 10% of the best achievable accuracy over the entire range of the parameter values evaluated across the data sets. Since there was no evidence to support changing the original Process P parameter values, they were retained in the present model.

#### A note on the phase response curve characteristics

Earlier versions of the limit-cycle oscillator models also reported the human PRC characteristics and how well the respective models predicted PRC data as published in Khalsa et al. (1997). Their analysis of the shift in the melatonin midpoint revealed a characteristic type 1 PRC with a significant peak-to-trough amplitude of 5.02 h.

Naturally, we wanted to investigate whether in trying to achieve the highest accuracy for predicting phase change for the four data sets, there was no inadvertent compromise to the accuracy of predicting the PRC.

**FIGURE 5:**
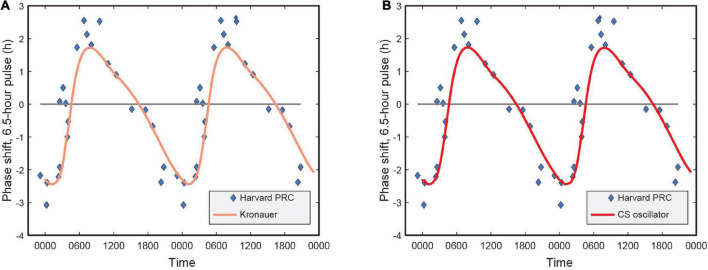
**(A)** Phase response curve for the Kronauer model with photopic illuminance as light stimulus input; **(B)** Phase response curve using CS as light stimulus input. CS = circadian stimulus.

Our analysis revealed that a re-characterization of the light stimulus input from lux to CL_A_ (and then CS), without changing other parameters of the Kronauer99 framework, had no bearing on the PRC characteristics (see identical plots in Figure 5). This was expected, as the PRC predictions use a constant spectrum, intensity, and duration pulse of light for which only the time-of-day of exposure changes.

## Discussion

A model for predicting light-induced circadian phase changes can aid in the adjustment of circadian phase to support re-entrainment for shift work, military operations, and air travel. In this regard, a breakthrough was the van der Pol oscillator model developed by Kronauer et al. (1999, 2000). As we have shown in the present contribution, to date, no other model has made substantive improvements to its prediction accuracy. The present systematic examination of the Kronauer99 model showed that it remains as good as any in terms of prediction uncertainty, which is limited to about 1 h.

We approached the analysis of the Kronauer99 model using a well-established framework from experimental psychology whereby the stimulus (S) acts on the organism (O) to produce a response (R). Within that framework, using four independent data sets, we conducted a serial analysis of the factors in the Kronauer99 model that could affect prediction accuracy. This analysis led to several conclusions.

***First***, it has already been established that light processed by the retina is the most important phase-shifting stimulus (S) to the master clock in the SCN. However, the definition of light for stimulating the master clock in the Kronauer99 model relies upon photopic illuminance where the spectral weighting function is the photopic luminous efficiency function [V(λ)]. From a wealth of research conducted over the last 20 years, V(λ) is incorrect for characterizing the spectral sensitivity of the circadian system. Recent modeling efforts of circadian phototransduction (Rea et al., 2005, 2012, 2021a,2021b) have quantified the non-linear spectral sensitivity of circadian-effective light (CL_A_). Replacing photopic illuminance with irradiance at the cornea weighted by CL_A_ improved prediction accuracy.

*Conclusion:* The spectral sensitivity of the system is important.

***Second***, for satisfactory predictions, Kronauer99 introduced a compressive function of the raw photopic illuminance via Process L. This function has no link to retinal neurophysiology but was simply a convenient and necessary transformation needed to increase prediction accuracy. In addition to defining the spectral sensitivity, the functional relationship between optical irradiance incident on the cornea and the response of these retinal mechanisms has been quantified in terms of circadian stimulus (CS) from threshold to saturation (Rea and Figueiro, 2018; Rea et al., 2021a,b). CS represents an inherently compressive function of circadian-effective light based upon the neurophysiology of the retina. Therefore, the neural signal reaching the SCN defined in terms of CS is quantified in terms of both its spectral sensitivity and its operating characteristic. The CS transformation obviated arbitrary compression of the light input via I_0_ and improved the prediction accuracy. Derived from the Kronauer99 framework with revised characterization of the input photic stimulus in units of CS, the revised pacemaker model has been referred to as the CS-oscillator model.

*Conclusion:* The operating characteristic of retinal mechanisms providing input to the system is important.

***Third***, Kronauer99 introduced Process L to account for hysteresis by the circadian-phototransduction mechanisms. In effect, this means that the effectiveness of a given light pulse is not independent of the effectiveness of the previous light pulse. All neural systems, including those in the retina, adapt to repeated stimulation. Although the tie to retinal neurophysiology is weaker than that for spectral and absolute sensitivity to light (above), this non-linear response of the retina to light was shown to be critical for prediction accuracy.

C*onclusion:* The system adapts to successive light exposures.

***Fourth***, diurnal humans, on average, have an intrinsic period of slightly longer than 24 h. Thus, morning light will, in principle, advance the clock to keep entrainment. For the CS-oscillator model, we examined whether measuring morning light exposure alone would accurately predict phase changes. It was quite clear that it was necessary to collect *all* light data throughout the day to accurately predict phase changes.

*Conclusion:* Every photon counts.

***Fifth***, the time of light exposure is important. Even though a typical CBT_min_ value of 0400 appears to be a good estimate of the circadian phase marker for most people, individual differences are important. Most notably, the cohort from Sharkey et al. (2011) was a group of phase-delayed adolescents and young adults and a sensitivity modulator term of 0800 for CBT_min_ produced the most accurate predictions for that cohort.

*Conclusion:* An individual’s chronotype plays an important role in model predictions.

***Sixth***, after integrating these five conclusions into the CS-oscillator model, we examined the utility of the free-parameter coefficients in the original Kronauer99 formulation. We could find no compelling reason to adjust the original coefficients.

*Conclusion:* The original model by Kronauer99 and colleagues represents the best framework for predicting phase shifting following light exposure.

Table 8 shows how accurate the original Kronauer99 model was in predicting the four independent data sets used in the present analysis along with the improvements introduced here. In sum, the CS-oscillator model developed here provides improved prediction accuracy.

**TABLE 8:** Overall improvement in the prediction accuracy.

Model	Data set	*R* ^2^	Mean absolute error (MAE) in h	Subjects with error < 1.0 h (%)
Original Kronauer99	Average (3 studies)	0.25	1.07	53
CS-Oscillator*	Average (4 studies)	0.58	0.61	88

Note that the Appleman et al. (2013) data could not be used with the Kronauer99 model predictions.

*CS-Oscillator model assumes the optimum CBT_min_ as reported in Table 7.

These predictions are still not as good as one might hope. A new look at the phase predictions within the S-O-R framework is worthwhile. It is important to emphasize that models of the organism’s pacemaker (O) will not be successful without also including an understanding of the light stimulus (S) and the down-stream response (R). Ideally too, it would be important to tie any model developments to the neurophysiological and endocrinological mechanisms being modeled. This convergence of a mathematical model with the underlying biophysics would provide a much higher level of confidence in the model itself.

## Future research

The primary value of any model is to make quantitative predictions. The model developed here, based upon the earlier Kronauer99 model, provides the foundation for future steps and focused hypotheses for empirical testing related to the stimulus (S), response (R), and organism (O).

### Stimulus

A good understanding of the circadian phototransduction mechanisms in the retina providing the neural stimulus to the SCN has been proposed. This understanding led to CL_A_ and CS metrics which provided better prediction accuracy for the Kronauer99 model than photopic illuminance and I_0_. Light sensors based upon that understanding should be further developed. Ideally, small sensors mounted near the user’s corneas should provide excellent characterization of the light stimulus. A previous field study undertaken by our lab involving light measurements over five consecutive days from 12 healthy participants (>65 years) compared the performance characteristics of the Daysimeters worn at four different locations (wrist, spectacle/headset, pendant, pin on torso). That study revealed that light measurements from pendant and pin on torso locations closely matched those from the spectacle/headset location (Δ < 5%), and that the light measurements from wrist location were consistently lower compared (Δ∼20%) to those from the spectacle/headset location (Figueiro et al., 2013). Finally, sampling rates with these sensor systems should be high due to hysteresis by the neural mechanisms governing circadian phase.

### Response

Relying on a single downstream measure of circadian phase seems risky. DLMO may be the best outcome measure we have, but more unconfounded downstream measures of circadian phase will reduce that risk if they provide consistent results. Little is known, however, about the temporal consistency in the changes of different circadian phase markers in response to light exposure. The amplitude of a circadian rhythm, like melatonin concentration, may also be important for health outcomes. Shift workers, who are at greater risk for diseases like Type II diabetes (Knutsson and Kempe, 2014), usually exhibit their highest melatonin concentrations during the night, but the amplitude of their rhythms is much curtailed. Perhaps response measures of both phase and amplitude would enable more accurate predictions.

### Organism

The van der Pol oscillator appears to be a very good model for the behavior of the SCN. However, the Kronauer99 conceptualization of the oscillator may be incomplete. Perhaps the core and the shell of the SCN have different oscillators and are subject to different stimuli and different feedback mechanisms. Further, there are many peripheral clocks within the body (e.g., the retina or the liver). Little is known, however, about the governing principles for synchronizing the central and the peripheral clocks or how important feedback from peripheral clocks is to the central clock phase. Daan and Pittendrigh (1976) proposed a pacemaker framework comprising of two coupled, evening (E) and morning (M), oscillators. Flores and Oda (2020) investigated the effects of photoperiod on the E-M model using two coupled non-linear limit-cycle oscillators, governed by the Pittendrigh-Pavlidis equations. The framework for this dual-oscillator model, pertaining to organization of light input for the SCN, is however, a work in progress and not ready for field validation. Relatedly, and as shown here, individual chronotypes affect the sensitivity modulator, so measuring circadian phase on a subject-by-subject or patient-by-patient basis before and following a light intervention appears to be critical for predicting light-induced phase changes. Assuming a “typical” baseline CBT_min_ time for all individuals will lead to poor predictions, particularly for those of extreme chronotype.

## Data availability statement

The original contributions presented in this study are included in the article/Supplementary material, further inquiries can be directed to the corresponding author.

## Ethics statement

The studies involving human participants were reviewed and approved by Institutional Review Board at Rensselaer Polytechnic Institute, where all four studies employed in the analyses were conducted. The patients/participants provided their written informed consent to participate in this study.

## Author contributions

MR developed the theoretical framework and served as the primary author of the manuscript. RN performed the modeling exercise, developed the preliminary graphics and tables, and served as the secondary author of the manuscript. AB supervised the analytical approach and helped develop supplementary data. MF conceived the study plan, acquired the funding for the original idea, and provided the leadership in preparation of the manuscript. All authors contributed to the article and approved the submitted version.
