# Supplementary material for: The circadian stimulus-oscillator model: Improvements to Kronauer’s model of the human circadian pacemaker
Source: Front Neurosci. 2022 Sep 27;16:965525. doi: 10.3389/fnins.2022.965525 (PMC9552883; doi:10.3389/fnins.2022.965525)
Supplement: Supplementary file 1 [file Data_Sheet_1.pdf]

Supplementary Material

APPENDIX 1: Mathematical overview of the Rea et al. (Rea et al., 2021a; b) model of human circadian phototransduction

$$CL_A 2.0 = 1548 \begin{cases} \left(\int Mc_\lambda E_\lambda d\lambda - a_{rod1}\left(\frac{\int V'_\lambda E_\lambda d\lambda}{\int V_{c\lambda} E_\lambda d\lambda + g_1 \int S_{c\lambda} E_\lambda d\lambda}\right)\left(1 - e^{\frac{-\int V'_\lambda E_\lambda d\lambda}{RodSat}}\right)\right) \\ + \left(a_{b-y}\left(\int S_{c\lambda} E_\lambda \mathrm{d}\lambda - k \int V_{c\lambda} E_\lambda \mathrm{d}\lambda\right) - a_{rod2}\left(\frac{\int V'_\lambda E_\lambda d\lambda}{\int V_{c\lambda} E_\lambda d\lambda + g_2 \int S_{c\lambda} E_\lambda d\lambda}\right)\left(1 - e^{\frac{-\int V'_\lambda E_\lambda \mathrm{d}\lambda}{RodSat}}\right)\right), & b - y > 0 \\ \left(\int Mc_\lambda E_\lambda d\lambda - a_{rod1}\left(\frac{\int V'_\lambda E_\lambda d\lambda}{\int V_{c\lambda} E_\lambda d\lambda + g_1 \int S_{c\lambda} E_\lambda d\lambda}\right)\left(1 - e^{\frac{-\int V'_\lambda E_\lambda d\lambda}{RodSat}}\right)\right), & b - y \leq 0 \end{cases}$$

…(A1.1)

where,

$$b - y = \int S_{c\lambda} E_\lambda \mathrm{d}\lambda - k \int V_{c\lambda} E_\lambda \mathrm{d}\lambda$$

$k = 0.2616$

$a_{b-y} = 0.21$

$a_{rod1} = 2.30$

$a_{rod2} = 1.60$

$g_1 = 1.00$

$g_2 = 0.16$

$RodSat = 6.50\ W\ m^{-2}$

E_λ: light source spectral irradiance.
Mc_λ: melanopsin sensitivity (corrected for crystalline lens spectral transmittance) (Wyszecki and Stiles, 1982).
S_λ: S-cone fundamental (Smith and Pokorny, 1975).
mp_λ: macular pigment spectral transmittance (Snodderly et al., 1984).
V_λ: photopic luminous efficiency function (Commission Internationale de l'Éclairage, 1994).
V'_λ: scotopic luminous efficiency function (Commission Internationale de l'Éclairage, 1994).

$$V_{c\lambda} = \frac{\left(\frac{V_\lambda}{mp_\lambda}\right)}{\max\left(\frac{V_\lambda}{mp_\lambda}\right)} \qquad S_{c\lambda} = \frac{\left(\frac{S_\lambda}{mp_\lambda}\right)}{\max\left(\frac{S_\lambda}{mp_\lambda}\right)}$$

$$CS = 0.7 * \left[1 - \frac{1}{1 + \left(\frac{CL_A 2.0}{355.7}\right)^{1.1026}}\right]$$

…(A1.2)

APPENDIX 2: Mathematical overview of the Kronauer model

The original van der Pol oscillator proposed by Kronauer (1990) (Kronauer90) to model the pacemaker is given by equation A2.1:

$$\left(\frac{12}{\pi}\right)^2 \ddot{x} + \mu(-1 + 4x^2)\left(\frac{12}{\pi}\right)\dot{x} + \left(\frac{24}{\tau}\right)^2 x = 0$$

(A2.1)

The variable x represents an oscillating quantity within the pacemaker and the dot and double dot notation above x indicate the first and second derivative with respect to time, respectively. Hence, equation A2.1 is a second-order ordinary differential equation. Kronauer90 assigned the x variable to be proportional to endogenous core body temperature (CBT). The variable τ represents the intrinsic period of the pacemaker, 24.2 hours, and μ scales the nonlinear damping of the oscillator (confusingly called "stiffness" in Kronauer's papers perhaps because of its nonlinear polynomial multiplier). Positive damping removes energy from the oscillations resulting in smaller amplitude (i.e., smaller changes in body temperature), while negative damping adds energy and increases the amplitude of oscillation. This van der Pol oscillator is described as a self-sustaining limit-cycle oscillator because when left to stabilize from any starting condition the amplitude will settle at a balancing value (1 in this case) where larger values result in a net loss of energy (amplitude) via damping and negative values result in a net gain of energy (amplitude). The quadratic damping polynomial controls this characteristic (Figure A2.1).

To facilitate analysis and numerically solving for x as a function of time, equation A2.2 can be expressed as a set of two first-order differential equations by applying a Liénard transformation.

$$\dot{x} = \left(\frac{\pi}{12}\right)\left[x_c + \mu\left(x - \frac{4}{3}x^3\right)\right]$$

$$\dot{x}_c = -\left(\frac{\pi}{12}\right)\left(\frac{24}{\tau}\right)^2 x$$

…(A2.2)

x and x_c are two independent state variables. As above, x represents the endogenous CBT and x_c is a "complementary" variable and not assigned to any pacemaker outcome or attribute.

The effect of a light-induced stimulus on the pacemaker is modeled by adding a driving term, B, to each first-order state equation. The term 'B' is synonymous with the term 'S' or 'stimulus' as discussed in the article. The coefficient q controls how sensitive the system is to light when recovering from a low amplitude condition (equation A2.3).

$$\dot{x} = \left(\frac{\pi}{12}\right)\left[x_c + \mu\left(x - \frac{4}{3}x^3\right) + B\right]$$

$$\dot{x}_c = \left(\frac{\pi}{12}\right)\left[qBx_c - \left(\frac{24}{\tau}\right)^2 x\right]$$

…(A2.3)

Kronauer et al. revised this model in 1998 (Kronauer98) to better predict the recovery of the pacemaker following critically timed light exposures that bring the pacemaker amplitude near zero. This was done by replacing the damping with a higher order polynomial with less negative damping near zero, greater negative damping as the amplitude approaches 1, and then a rapid reversal to

positive damping for amplitudes close to and greater than 1 (see figure A2.1). This change slowed the recovery at small amplitudes and quickened the recovery at larger amplitudes.

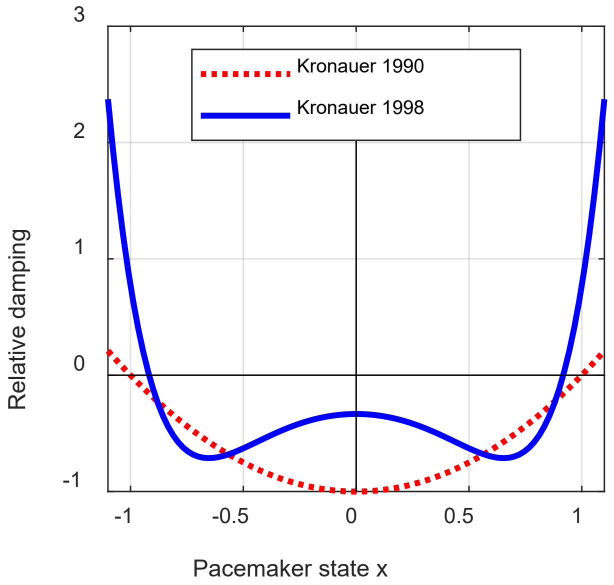

Figure A2.1. The Comparison of damping terms in Kronauer pacemaker models: quadratic damping in Kronauer90 and 6th order damping in the Kronauer98 and Kronauer99.

The first-order state equations for the Kronauer98 model are:

$$\dot{x} = \left(\frac{\pi}{12}\right)\left[x_c + \mu\left(\frac{1}{3}x + \frac{4}{3}x^3 - \frac{256}{105}x^7\right) + B\right]$$

$$\dot{x}_c = \left(\frac{\pi}{12}\right)\left[qBx_c - \left(\frac{24}{\tau - 0.064}\right)^2 x - kBx\right]$$

…(A2.4)

The damping polynomial coefficients were adjusted to maintain a free-running amplitude of 1 and a small correction was subtracted from τ to negate the effect that the high-order damping has on the period. An additional light drive term was added to the x_c equation to better predict unpublished phase response curve data near the cross-over point at minimum CBT. The initial *direct-drive* pacemaker models (Kronauer, 1990; Jewett and Kronauer, 1998), wherein light (B) exerts a direct influence on the state variables of the oscillator, could only accurately describe the response of the human circadian system to extended (4-8 h) bright (~10,000 lux) light stimuli. In 1999 and 2000, Kronauer and colleagues (Kronauer et al., 1999; 2000) introduced the concept of a dynamic stimulus processor (Process L) in the model (Kronauer99) that intervenes between the S and its effect on the self-sustaining limit-cycle oscillator (Process P), to allow predictions of R without limiting predictions to the lighting conditions from the original experiments. Process L is given by the following equations.

$$\dot{n} = 60[\alpha(1-n) - \beta n] \qquad \dots \quad (A2.5)$$

$$\alpha = \alpha_o \left(\frac{I}{I_o}\right)^p \qquad \dots \quad (A2.6)$$

$$\hat{B} = G\alpha(1-n) \qquad \dots \quad (A2.7)$$

$$\alpha_o = 0.05\ \text{min}^{-1}, \beta = 0.0075\ \text{min}^{-1}, G = 33.75, I_o = 9500\ lux,\ p = 0.5$$

The state variable n controls how strongly the system responds to light. Kronauer99 describe it as the fraction of photoreceptor elements available in the ready state at any given time. Light exposure converts ready receptor elements to the used state and over time the used elements are regenerated back to the ready state. The result is reduced effectiveness of sustained light exposures. Process L is controlled by two time constants: α_0 controlling the falloff of sensitivity with habituation and β controlling the recovery rate. An example of the effect of the L-process on a light stimulus is shown in Figure A2.2.

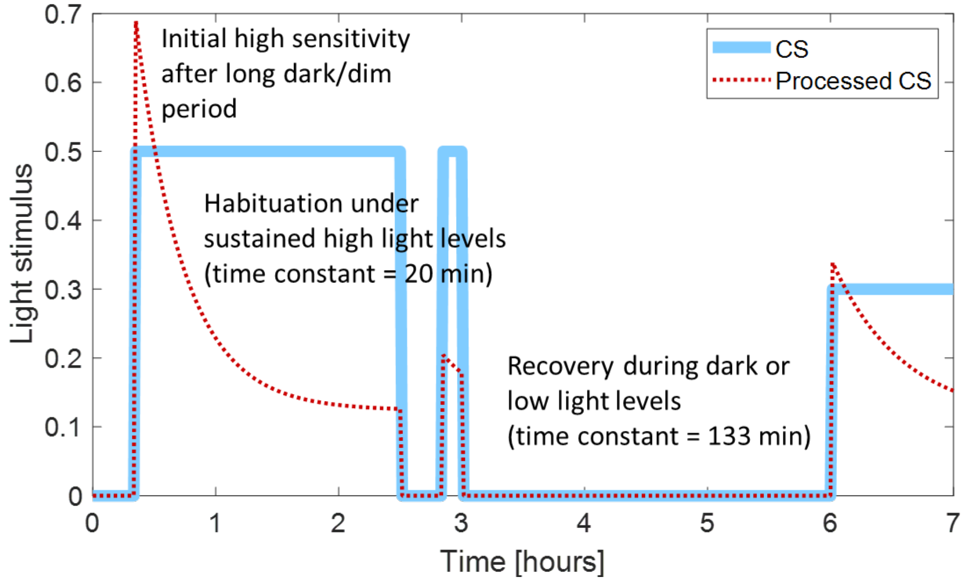

Figure A2.2. The Exemplar light stimulus (CS) and the resulting L-process conditioning of the signal before relaying it to the pacemaker P-process.

A sensitivity modulator was also added to vary the effectiveness of light exposure over the circadian day. The equation for this modulator uses the pacemaker state variables for timing as developed by Jewett et al. (1999). The modulator is inserted between the L- and P-processes.

$$B(t) = (1 - 0.4x)(1 - 0.4x_c)\hat{B}(t) \qquad \dots (A2.8)$$

A graphical description of the Kronauer99 framework is shown in Figure A2.3.

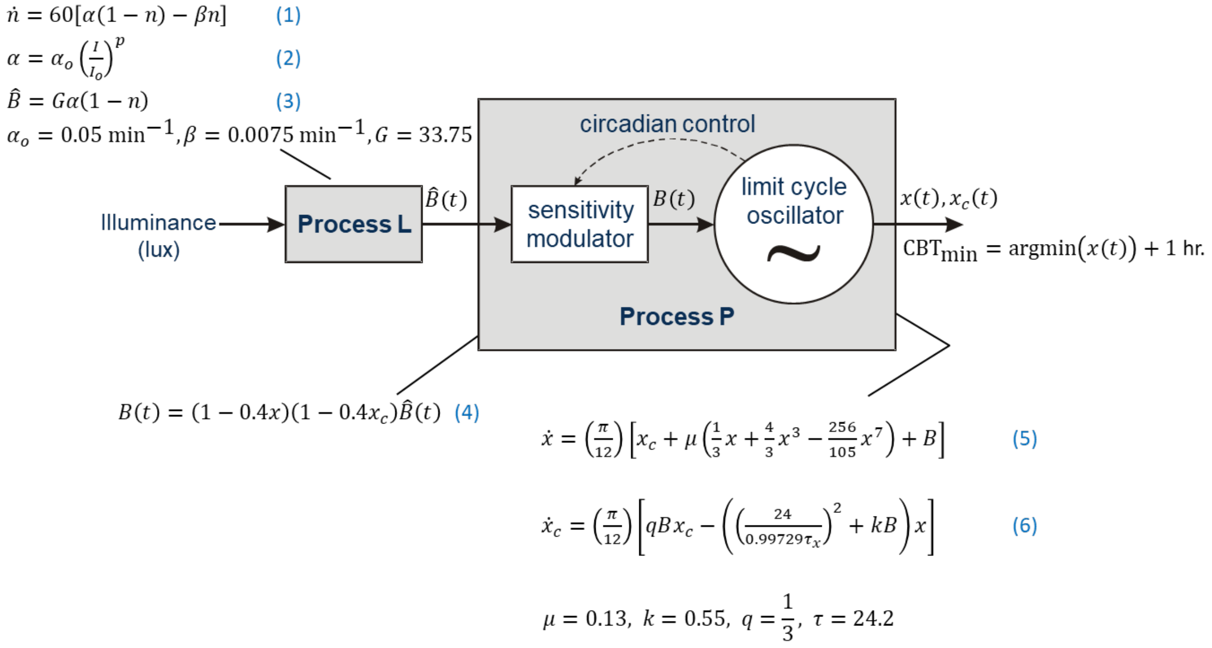

Figure A2.3. Graphical depiction of the Kronauer99 framework (Kronauer et al., 1999).

APPENDIX 3: Comparing predictions for the Kronauer99 model and the Forger99 model to determine the baseline prediction accuracy

Summary of model predictions for the Kronauer99 and the Forger99 models

Model	Dataset	R^2	Mean absolute error (MAE) in h	% subjects with error < 1.0 h
Kronauer99	Rea et al., (2016)	0.11	0.91	55%
	Figueiro et al. (2014)	0.42	0.86	67%
	Sharkey et al. (2011)	0.21	1.43	36%
	Average	0.25	1.07	53%
Forger99	Rea et al. (2016)	0.12	0.91	64%
	Figueiro et al. (2014)	0.39	0.89	62%
	Sharkey et al. (2011)	0.20	1.28	36%
	Average	0.24	1.03	54%

The average MAE of 1.07 h in predicting ΔDLMO across the three datasets (Sharkey et al., 2011; Figueiro et al., 2014; Rea et al., 2016) using the original Kronauer99 model was quite comparable to the average MAE of 1.03 h for the Forger99 model. The modeling exercise further revealed that the percentage subjects with error < 1 h was 53% and 54% for the Kronauer99 and Forger99 models, respectively.

APPENDIX 4: Effect of changing L-process parameters on MAE across the four datasets (Sharkey et al., 2011; Appleman et al., 2013; Figueiro et al., 2014; Rea et al., 2016) for the CS-oscillator model

Data set	a_0 and β scatter	p
Rea et al. (2016)	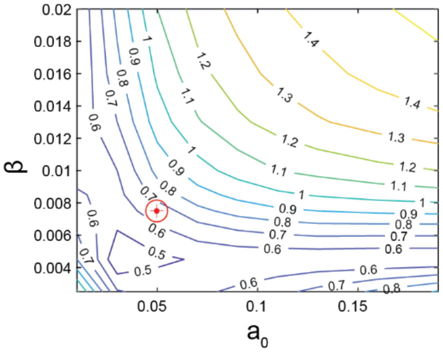	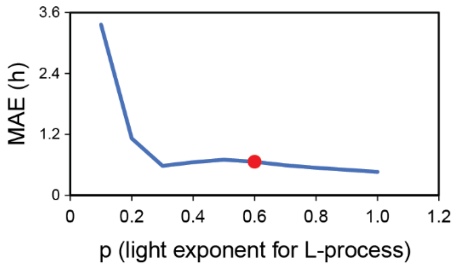
Figueiro et al. (2014)	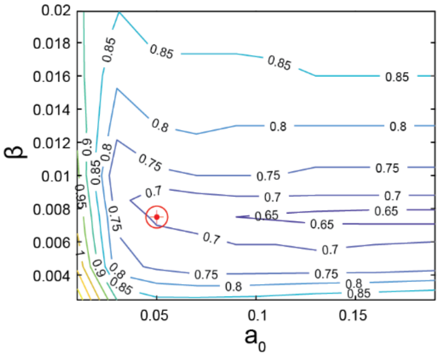	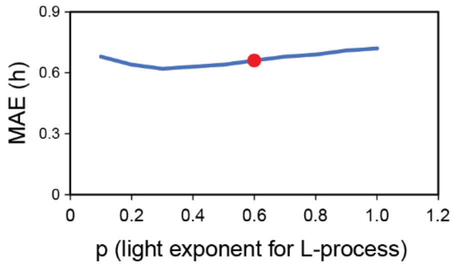
Sharkey et al. (2011)	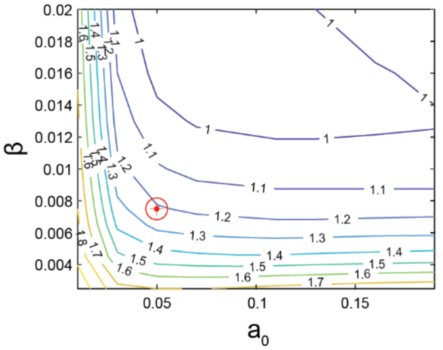	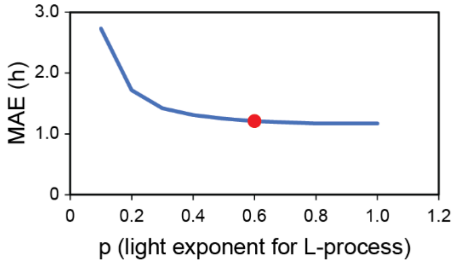
Appleman et al. (2013)	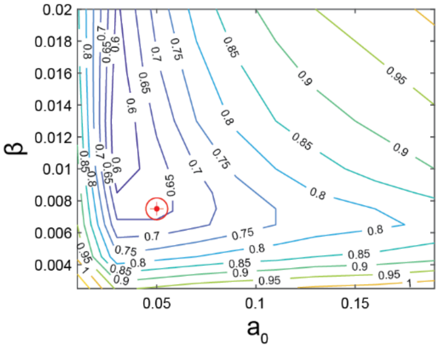	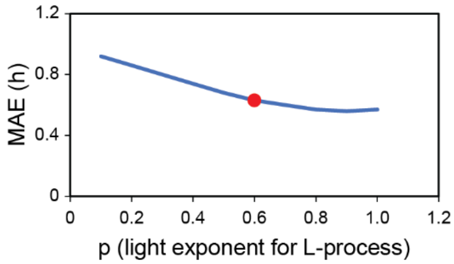

Note: The red targets (second column) and filled circles (third column) depict the prediction accuracy for Kronauer99 published values; * base case $p = 0.6$.

APPENDIX 5: Effect of changing L-process parameters on percentage subjects with error < 1 h across the four datasets for the CS-oscillator model

Data set	a_0 and β scatter	p
Rea et al. (2016)	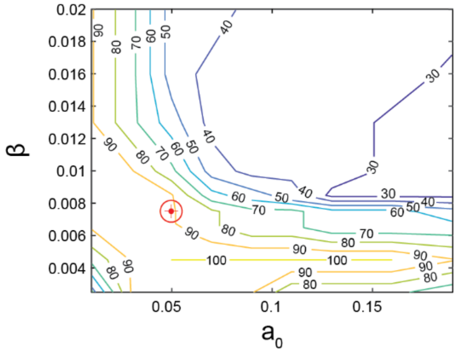	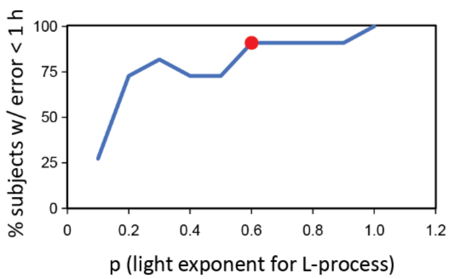
Figueiro et al. (2014)	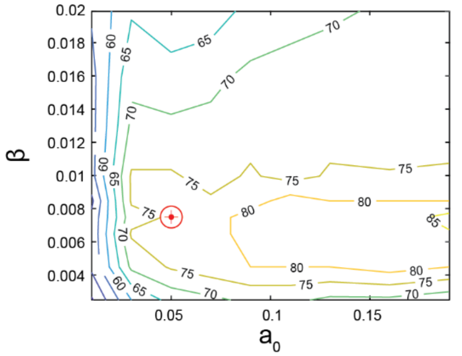	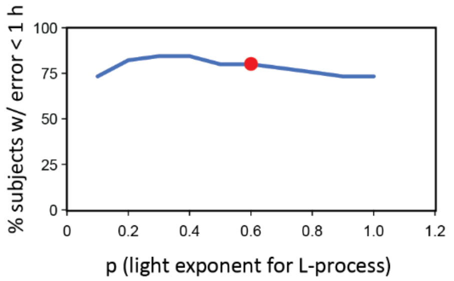
Sharkey et al. (2011)	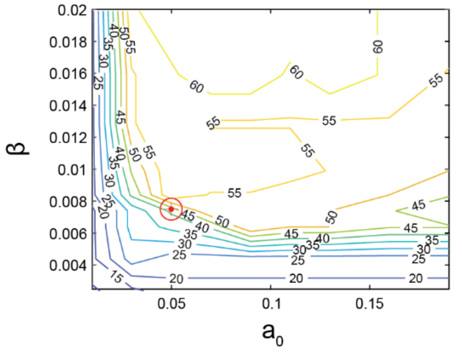	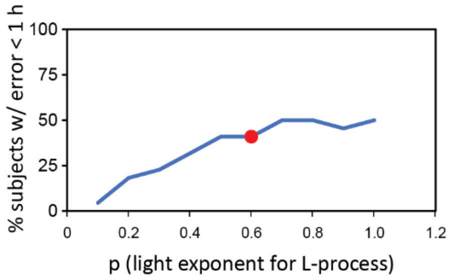
Appleman et al. (2013)	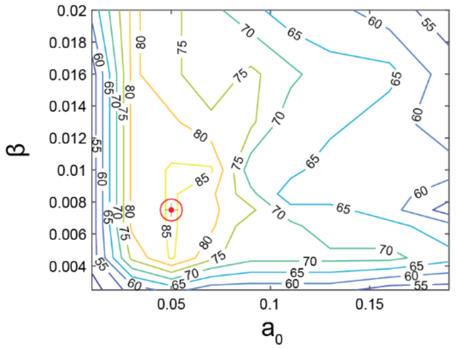	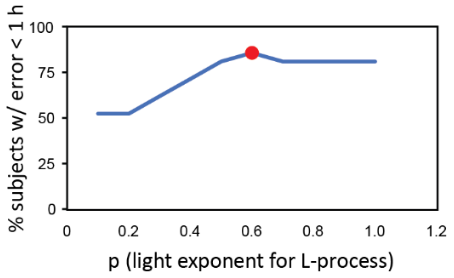

Note: The red targets (second column) and filled circles (third column) depict the prediction accuracy for Kronauer99 published values; * base case $p = 0.6$.

APPENDIX 6: Effect of changing P-process parameters on MAE across the four datasets (Sharkey et al., 2011; Appleman et al., 2013; Figueiro et al., 2014; Rea et al., 2016) for the CS-oscillator model

Data set	μ	k	q
Rea et al. (2016)	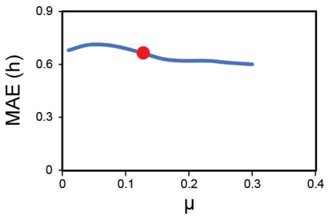	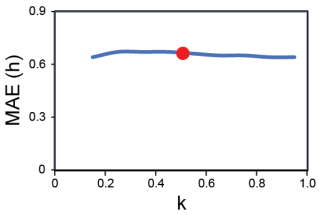	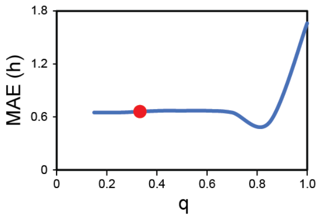
Figueiro et al. (2014)	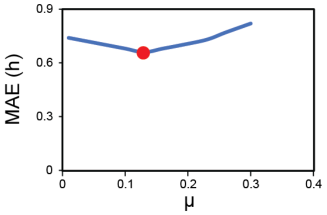	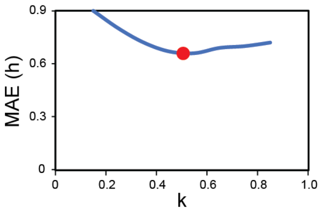	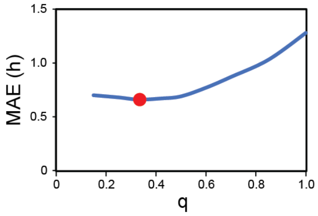
Sharkey et al. (2011)	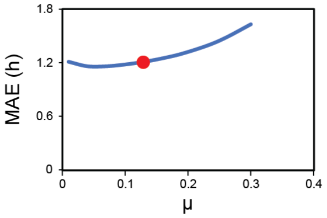	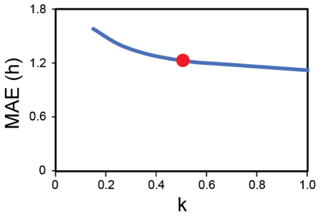	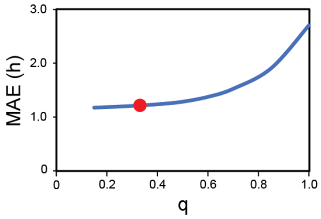
Appleman et al. (2013)	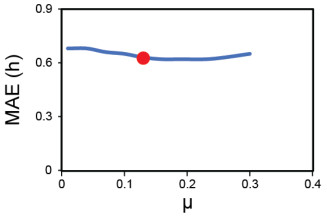	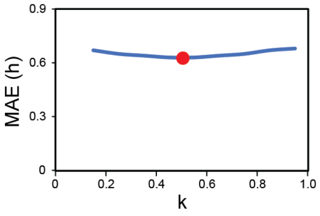	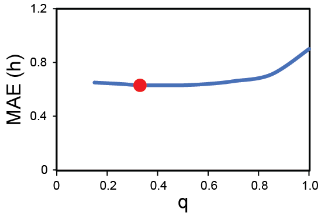

Note: The red filled circles depict the prediction accuracy for Kronauer99 published values; * base case $p = 0.6$.

APPENDIX 7: Effect of changing P-process parameters on percentage subjects with error < 1 h across the four datasets (Sharkey et al., 2011; Appleman et al., 2013; Figueiro et al., 2014; Rea et al., 2016) for the CS-oscillator model

Data set	μ	k	q
Rea et al. (2016)	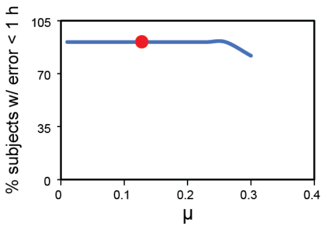	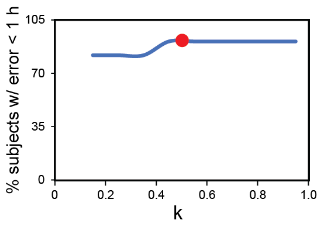	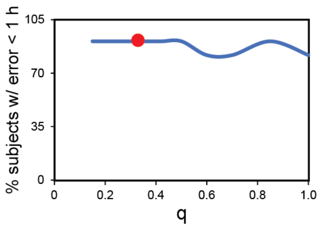
Figueiro et al. (2014)	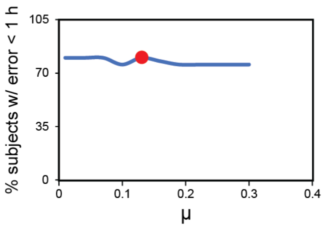	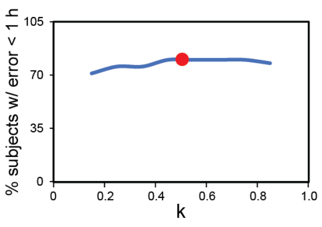	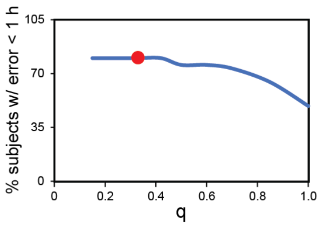
Sharkey et al. (2011)	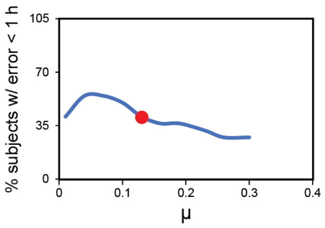	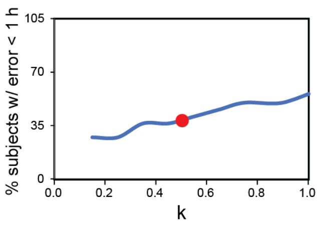	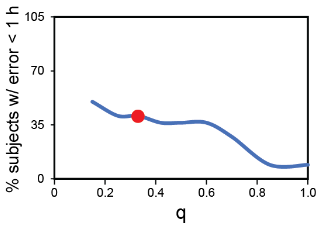
Appleman et al. (2013)	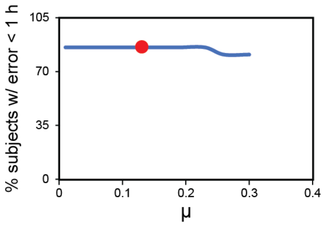	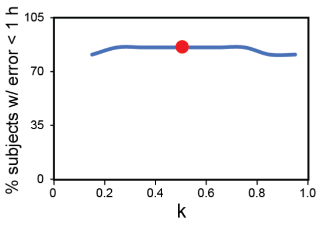	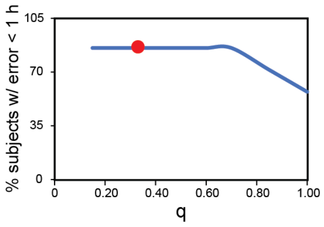

Note: The red filled circles depict the prediction accuracy for Kronauer99 published values; * base case p = 0.6.

REFERENCES

Appleman, K., Figueiro, M.G., and Rea, M.S. (2013). Controlling light-dark exposure patterns rather than sleep schedules determines circadian phase. *Sleep Med.* 14(5)**,** 456-461. doi: 10.1016/j.sleep.2012.12.011.

Commission Internationale de l'Éclairage (1994). "Light as a True Visual Quantity: Principles of Measurement". (Vienna: Commission Internationale de l'Éclairage).

Figueiro, M.G., Plitnick, B., and Rea, M.S. (2014). The effects of chronotype, sleep schedule and light/dark pattern exposures on circadian phase. *Sleep Med.* 15(12)**,** 1554–1564. doi: 10.1016/j.sleep.2014.07.009.

Jewett, M.E., Forger, D.B., and Kronauer, R.E. (1999). Revised limit cycle oscillator model of human circadian pacemaker. *J. Biol. Rhythms* 14(6)**,** 493-499. doi: 10.1177/074873049901400608.

Jewett, M.E., and Kronauer, R.E. (1998). Refinement of a limit cycle oscillator model of the effects of light on the human circadian pacemaker. *J. Theor. Biol.* 192(4)**,** 455-465. doi: 10.1006/jtbi.1998.0667.

Kronauer, R.E. (1990). "A quantitative model for the effects of light on the amplitude and phase of the deep circadian pacemaker, based on human data," in Sleep '90, Proceedings of the 10th ESRS Congress*,* ed. J. Horne. (Bochum, Germany: Pontenagel Press), 306-309.

Kronauer, R.E., Forger, D.B., and Jewett, M.E. (1999). Quantifying human circadian pacemaker response to brief, extended, and repeated light stimuli over the phototopic range. *J. Biol. Rhythms* 14(6)**,** 500-516. doi: 10.1177/074873049901400609.

Kronauer, R.E., Forger, D.B., and Jewett, M.E. (2000). Erratum to: Quantifying human circadian pacemaker response to brief, extended, and repeated light stimuli over the photopic range. *J. Biol. Rhythms* 15(2)**,** 184-186.

Rea, M.S., Nagare, R., and Figueiro, M.G. (2021a). Modeling circadian phototransduction: Quantitative predictions of psychophysical data. *Front. Neurosci.* 15**,** 44. doi: 10.3389/fnins.2021.615322.

Rea, M.S., Nagare, R., and Figueiro, M.G. (2021b). Modeling circadian phototransduction: Retinal neurophysiology and neuroanatomy. *Front. Neurosci.* 14**,** 1467. doi: 10.3389/fnins.2020.615305.

Rea, M.S., Plitnick, B., and Figueiro, M.G. (2016). Effect of custom blue light intervention on dim light melatonin onset in healthy adults. (Unpublished manuscript). Troy, NY: Lighting Research Center, Rensselaer Polytechnic Institute.

Sharkey, K.M., Carskadon, M.A., Figueiro, M.G., Zhu, Y., and Rea, M.S. (2011). Effects of an advanced sleep schedule and morning short wavelength light exposure on circadian phase in young adults with late sleep schedules. *Sleep Med.* 12(7)**,** 685-692. doi: 10.1016/j.sleep.2011.01.016.

Smith, V.C., and Pokorny, J. (1975). Spectral sensitivity of the foveal cone photopigments between 400 and 500 nm. *Vision Res.* 15**,** 161-171. doi: 10.1016/0042-6989(75)90203-5.

Snodderly, D.M., Brown, P.K., Delon, F.C., and Auran, J.D. (1984). The macular pigment: I. Absorbance spectra, localization, and discrimination from other yellow pigments in primate retinas. *Invest. Ophthalmol. Vis. Sci.* 25(6)**,** 660-673.

Wyszecki, G., and Stiles, W.S. (1982). *Color Science: Concepts and Methods, Quantitative Data and Formulae.* New York, NY: John Wiley & Sons.
